# Machine Learning–Based Screening of Healthy Meals From Image Analysis: System Development and Pilot Study

**DOI:** 10.2196/18507

**Published:** 2020-10-26

**Authors:** Kyoko Sudo, Kazuhiko Murasaki, Tetsuya Kinebuchi, Shigeko Kimura, Kayo Waki

**Affiliations:** 1 Department of Information Sciences Toho University Chiba Japan; 2 NTT Media Intelligence Laboratories Yokosuka Japan; 3 Department of Ubiquitous Health Informatics Graduate School of Medicine, The University of Tokyo Tokyo Japan; 4 Department of Biomedical Informatics Graduate School of Medicine, The University of Tokyo Tokyo Japan

**Keywords:** meal images, healthiness, deep neural network, nutrition, medical informatics, diet, neural network

## Abstract

**Background:**

Recent research has led to the development of many information technology–supported systems for health care control, including systems estimating nutrition from images of meals. Systems that capture data about eating and exercise are useful for people with diabetes as well as for people who are simply on a diet. Continuous monitoring is key to effective dietary control, requiring systems that are simple to use and motivate users to pay attention to their meals. Unfortunately, most current systems are complex or fail to motivate. Such systems require some manual inputs such as selection of an icon or image, or by inputting the category of the user’s food. The nutrition information fed back to users is not especially helpful, as only the estimated detailed nutritional values contained in the meal are typically provided.

**Objective:**

In this paper, we introduce healthiness of meals as a more useful and meaningful general standard, and present a novel algorithm that can estimate healthiness from meal images without requiring manual inputs.

**Methods:**

We propose a system that estimates meal healthiness using a deep neural network that extracts features and a ranking network that learns the relationship between the degrees of healthiness of a meal using a dataset prepared by a human dietary expert. First, we examined whether a registered dietitian can judge the healthiness of meals solely by viewing meal images using a small dataset (100 meals). We then generated ranking data based on comparisons of sets of meal images (850 meals) by a registered dietitian’s viewing meal images and trained a ranking network. Finally, we estimated each meal’s healthiness score to detect unhealthy meals.

**Results:**

The ranking estimated by the proposed network and the ranking of healthiness based on the dietitian’s judgment were correlated (correlation coefficient 0.72). In addition, extracting network features through pretraining with a publicly available large meal dataset enabled overcoming the limited availability of specific healthiness data.

**Conclusions:**

We have presented an image-based system that can rank meals in terms of the overall healthiness of the dishes constituting the meal. The ranking obtained by the proposed method showed a good correlation to nutritional value–based ranking by a dietitian. We then proposed a network that allows conditions that are important for judging the meal image, extracting features that eliminate background information and are independent of location. Under these conditions, the experimental results showed that our network achieves higher accuracy of healthiness ranking estimation than the conventional image ranking method. The results of this experiment in detecting unhealthy meals suggest that our system can be used to assist health care workers in establishing meal plans for patients with diabetes who need advice in choosing healthy meals.

## Introduction

Recently, many information technology–supported systems for health care have been developed, including systems using image-based dietary assessment for obesity and diabetes management [1]. A survey of popular nutrition-related mobile apps demonstrated that there is clear interest for diet monitoring and recommendation using mobile apps [2]. With the inclusion of a camera feature, mobile devices are increasingly being used for image-based dietary assessment. One of these systems is DialBetics [3], which is assisted by the food image recognition app FoodLog [4] to input photos of meals by semiautomatically limiting the dish-selection area. Herein, we refer to a “meal” as the dish or dishes eaten by a person in a single sitting; thus, a single dish or a multiple-dish menu may constitute a meal. A narrative review of this system indicated that most patients actively used the dietary evaluation module, and each meal’s nutritional balance sent to the patients helped them to modify their diet. However, the image processing was used only to assist inputting meal photos and identifying the name of meals; the meals’ total energy, macronutrients, dietary fiber, and salt were calculated by dietitians from the photos.

However, two problems can cause people to stop using the system. The first problem is that most current technologies require user action to achieve meal image recognition [4,5]. Estimating nutrition automatically from only inputting meal images by users is expected to be an important function of information technology–supported systems for health care control. However, this has not been achieved by most of these systems, including DialBetics [3]. For instance, with DialBetics, the user may need to manipulate images so that only one dish is included, or may need to identify the food area within the broader plate area. The user generally must select the food category among those suggested by the system, and when some foods do not match any of the available categories, users must register a new category in the system. These fairly difficult tasks lead some users to discontinue use of the system [1,3].

The second problem is the disconnect between the system’s output and the user’s understanding. The interviews with users of Dialbetics showed that some users consider the advice obtained from the dietitian to be too long and redundant [3]. It is difficult for users to know whether their meals are good for them based on a simple listing, however detailed, of nutritional values. Therefore, a system is needed that offers immediate feedback allowing users to understand the nutritional implications of what they are eating and motivates them to become interested in eating better meals.

It seems to be a safe assumption that a “meal image,” a single image of all of the dishes in a meal, contains some visual clues that permit estimation of the healthiness of the meal. Using meal images, we aim to provide a simple interface between the user and the system, with clear feedback that motivates users to continue to care about their meals, and a way to screen those who need the advice of a registered dietitian to improve their meals. A previous study of a system that allows sharing meal feedback from other users based on images [6] showed that the feedback, even from users rather than experts, is effective in improving diet. Our system is based on a similar concept. The feedback is not detailed numeric nutrition data but rather a more intuitive rating that can be easily understood by users. We targeted health promotion and diabetes, since the basic conditions of ideal meals in these contexts can be shared, including adequate energy and balanced nutrition, avoidance of salt, and obtaining abundant dietary fiber. We also designed the system to assess one meal eaten at a time, since the guidelines of a healthy diet for patients with diabetes in Japan suggest having three meals a day, and ensuring a nutritionally balanced and equalized portion in each meal as much as possible [7].

Many food image recognition systems have been proposed in recent years [5,8-20]. The most popular approach uses general object recognition technologies that sort foods into categories. The development of machine learning and a large database have accelerated growth in the number of food categories that can be recognized in recent years [17-19]. One approach to nutrition estimation is category-based estimation; that is, recognizing the category of food and displaying the nutritional value of that category. This approach does not assess the amount of food, and value outputs assume that one dish contains a regular portion or requires users to select among a lineup of values for multiple people or photos of different portion sizes [14]. To estimate the amount of food in a given dish, each food area must be segmented and the volume of each segment estimated. Other recent approaches include one based on a convolutional neural network (CNN) that trains a model using large sets of food images and their nutritional values. To estimate nutrition more accurately, one approach simultaneously posits the name of the meal and the nutrition of that food [9,11], and another estimates the ingredients in each dish and their proportions/weights from which it calculates nutritional value [15,20]. Clearly, these food recognition systems based on machine learning require a large amount of training data. Recent research has involved food recognition with an original food dataset containing hundreds of food categories [17-19]. Nevertheless, given the extremely high number of different categories of food in the world, covering all of them with such a model is virtually impossible. Furthermore, home cooking often involves dishes that are hard to assign to an appropriate category name, making it difficult to conduct training. To identify food categories, some approaches recognize foods by their ingredients [12,13]. These approaches are valid when the appearance of the ingredients changes minimally during cooking. However, in most cases, the appearance will change greatly after cooking, making the scope of this approach rather limited.

In contrast to these conventional methods that estimate nutrition by recognizing the category of foods or ingredients, we propose a novel approach of predicting whether or not a meal is healthy by viewing the total meal image. Our approach avoids the highly difficult food image recognition task that determines the absolute values of foods. Instead, our healthiness estimation method uses a ranking network and the ranking data generated by comparing many pairs of meal images. We fuse a recognition network, trained with a food dataset and with masks of food areas, with a ranking network. This approach allows for extracting food features from meal images that contain multiple dishes, enabling rendering an accurate judgement about the entire meal. Here, we report the results of a pilot study to highlight the pipeline of our proposed system from generating the ranking data of healthiness of meals to unhealthy meal detection.

The machine-learning algorithms that can be used for comparisons among data are collectively referred to as machine-learning rank algorithms (MLRAs). Several MLRAs based on support vector machine (SVM) or CNN algorithms have been proposed. CNN is known to offer high performance in recognition tasks, and CNN-based rank algorithms have been applied to estimate various attributes of images. One study demonstrated the estimation of town attributes from urban images of landscapes [21]. We employed the same MLRA, and trained its CNN using meal images manually ranked in terms of healthiness. One difference between learning the rank of landscape images and learning the rank of meal images is that the healthiness of meals should be estimated solely from the foods or ingredients. Accordingly, meals that contain the same ingredients but are photographed on different plates should be estimated to have the same healthiness.

It is also important to prevent the network from learning relationships between healthiness and factors other than the food itself. Toward this end, our ranking network was structured to estimate rank using only the food area by learning using spatially selected areas, implemented using a mask of the region of interest in the training phase. Our network was then trained by many ordered image pairs, according to the ranking method of Dubey et al [21] or TrueSkill [22].

Although a network could be trained online, we adopted an offline approach for learning. We first established a ranking dataset, and then input pairs of images from the dataset when training the network. We used this approach because the ranking data are needed to calculate the deviation value of the ranking score, which is the output of our system that intuitively expresses the healthiness of meals. Our database was annotated not by crowdsourcing but rather by an expert (a registered dietitian). Establishing the ranking dataset in advance is helpful for the expert, which allows them to work at any convenient time and can redo the work if warranted. Once establishing the ranking data, we simply generated the sets of pairs of rankings from this dataset.

Rank-SVM [23,24], an MLRA-based approach, is also used for image retrieval. In the model proposed by Joachims [23], the rank of images was learned using a ranking dataset that had a few hundred images per category. In view of the limited size of our dataset, there is a possibility that Rank-SVM would perform better than CNN; therefore, we compared the performance of ranking score–based CNN to Rank-SVM in this experiment to optimize the system.

## Methods

### Workflow

**Figure 1:**
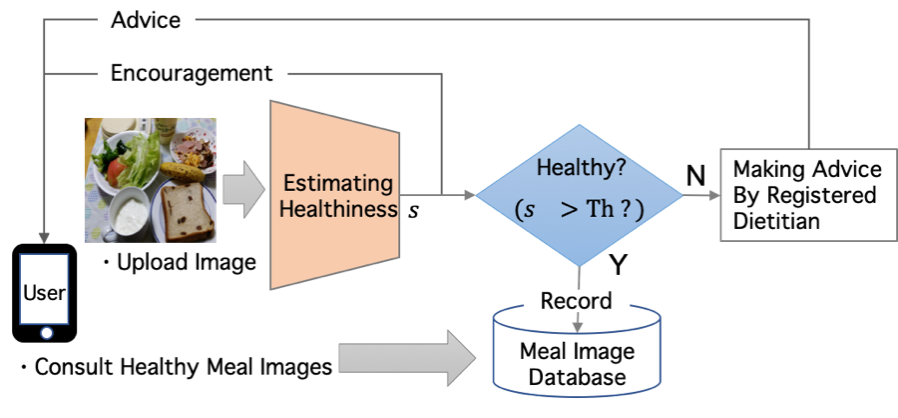
Workflow of the screening system and the image database of healthy meals for those who need the advice of a registered dietitian to improve their meals.

We propose an image-based system that can rank meals in terms of the overall healthiness of the dishes constituting the meal. First, we generated a database of meal images ranked by a registered dietitian viewing the images. We then constructed a network that maintains conditions important for judging the meal image, while extracting features that eliminate background information and those that are independent of location. The output of the network, *healthiness,* and the dietitian’s judgment were expected to be related with a high correlation coefficient. The workflow of our system is schematically shown in Figure 1.

Our system extracts the *healthiness score*, which is calculated as the deviation from average healthiness, so that users can know whether or not their meal is a healthy choice within the distribution of healthiness of meals by assessing how far they are from the average healthiness. In Figure 1, *s* is the healthiness score estimated by the proposed method and *Th* is the threshold of healthiness in screening dishes. When the healthiness score is high, the system sends the user the score and a message encouraging them to continue consuming healthy meals and using the system. The system further allows the users to check their records of the meal images with their healthiness scores; this function serves as a reference for the users to choose healthier meals. When the healthiness score is low (*s*<*Th*), a registered dietitian intervenes to help the user modify their diet. These users can consult the database for healthy meal images to improve their meals. Because the users obtain feedback immediately after they record their meals, they can change to a healthier meal if their initial choice achieves a low score.

### Framework Overview

Our image-based meal rating system performs the following processes: (1) examines whether a registered dietitian can judge the healthiness of meals solely by viewing meal images in a small dataset (100 meals); (2) generates ranking data based on comparisons of sets of meal images (850 meals) by a registered dietitian viewing meal images; (3) trains a network (a feature-extraction subnetwork, pretrained by a food dataset before being trained by our ranking dataset, and a ranking estimation subnetwork) based on the ranking to estimate a health metric; and (4) estimates each meal’s score, and the domain adaptability of estimating each meal’s score to finally detect unhealthy meals based on the health metric.

### Ranking Meal Healthiness

#### Ground Truth and Dietitian’s Subjective Evaluation

Ground truth consists of the sets of images and their associated ranks. The ground truth can be developed through cooking sample meals. The registered dietitian cooks the sample meals using ingredients with measured nutritional values to allow for accurate calculation of the total nutritional values using proven nutrition formulas [7]. The meal is then photographed and the set of nutritional values is ranked, resulting in a set of images and ranks.

This methodology is resource-intensive, and in practical situations leads to small datasets due to resource constraints. Therefore, we required larger datasets to train the network. Accordingly, we expanded the ground truth set using experts to perform subjective assessments of the healthiness rank based on images alone. To establish the validity of this approach, we verified that the expert, a registered dietitian, can appropriately judge the healthiness rank by viewing a meal image. We asked the dietitian to examine the images in the ground truth set and to rank them according to their nutritional value. The dietitian who judged the meal images was different from the dietitian who cooked the sample meals for the ground truth dataset and photographed them. We then appraised the relationship between the rank given by the dietitian viewing images of the meals and the ground truth rank based on nutritional measurements. The ground truth rank based on nutritional measurements was calculated according to the standard values of food composition in Japan [25,26]. Briefly, total energy and the energy ratio (ie, the ratio of protein, fat and carbohydrate), and the supplementary items (appropriateness of salt, and the amount vegetables, beans, and foods rich in dietary fiber such as mushrooms) are the items required for the calculation.

#### Generating Ranking Data

**Figure 2:**
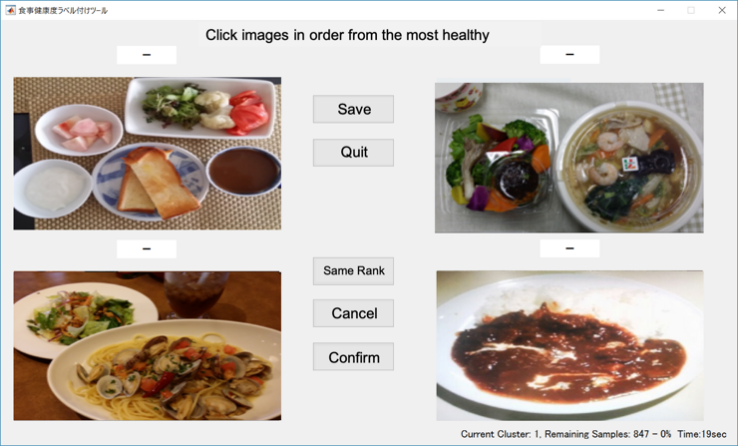
App interface to generate the ranking data. A set of 4 meal images is presented, and the registered dieticians rank those images from the 1st to 4th.

To generate a dataset containing the images and the healthiness metric implicit in each image, we used a custom app displaying a set of multiple meal images that were ranked by dietitians according to healthiness from “best” to “worst.” A dietitian was told that ranking should indicate whether the dishes could appropriately constitute an entire meal, and was also told to make the judgment as precisely as possible from only viewing the image of each meal. If it was difficult to differentiate between meals, the same rank assigned to multiple meals was acceptable. To reduce the dietitian’s workload, our app displays only 4 images at each step. The app interface is shown in Figure 2. For this study, we used the meal image database of patients with type 2 diabetes.

The ranking algorithm is shown in Multimedia Appendix 1, in which *N_c_*=4. We generated ranking data by repeatedly ranking sets of multiple meal images consisting of one pivot image and other images, according to step 17 in the algorithm, conducted by the dietitian. To generate a larger ranking dataset in the future, the TrueSkill [22] algorithm can be used, which considers the distribution of annotation that merges the rank sets annotated by multiple registered dietitians.

#### Proposed Network and Train Ranking

We trained the network to output the right ranking order of randomly selected pairs of images using the approach proposed by Dubey et al [21]. This approach was originally proposed for training urban images, where ranking was associated with safety, features were extracted from the whole image, and the score was output as a scalar value. Therefore, we had to adapt this algorithm to meal images.

The healthiness of the meals should be estimated solely from the comprising foods or ingredients. Therefore, meals that contain the same ingredients but were photographed on different backgrounds or plates should be estimated to have the same healthiness. To assure this continuity, we modified the ranking layer so that pixel features were only those included in the region of interest, eliminating any possible impact of food placement or background such as tables. In the training phase, we prevented the network from learning relationships between healthiness and factors other than the food itself by using a mask to indicate the region of food. The mask simply used a 1 value in the food area and a 0 value elsewhere, so that pixel-by-pixel multiplication of the original image and the mask resulted in an image where the food area is the same as the original image and any part of the image outside of the food area is given a value of 0. The mask was generated manually, outside the proposed system. The mask is applied only when the data are used for training; once the network is trained, masking is no longer necessary.

The network to predict healthiness was trained using the expanded ground truth ranking data generated by the registered dietitians. Pairs of images and their relative rankings were input in the training process, and all pairs were labeled to indicate which is healthier than the other. Duplicate networks were used to predict healthiness. Their outputs were used to calculate the loss, defined as:


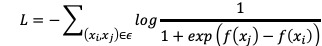
 **(1)**


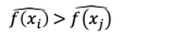
 (**2)**

where *x_i_, x_j_* is the pair of images *i*, *j* for training, ∈ is the set of all pairs of labeled images, and *f(x)* is the estimated healthiness of image *x*. 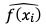 and 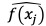 are the ground truth healthiness, and equation (2) is the relation between the ground truth order of images *x_i_* and *x_j_*.

We assign *i* and *j* for a pair of images as they satisfy 
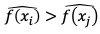
. Since the value of the loss function of equation (1) becomes smaller when the order of the estimated ranks *f(x_i_)* and *f(x_j_)* is *f(x_i_)*>*f(x_j_)* in the condition that the ground truth order relation between images *i* and *j* is 
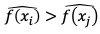
, the ranking predictor is trained so as to minimize the loss for all data. When *f(x_i_)*>*f(x_j_)*, it is true, and *L* will give a value close to 0. When the order of the estimated rank *f(x_i_)* and *f(x_j_)* is *f(x_i_)*<*f(x_j_)*, it is false, and *L* will give a large value.

As the network for feature extraction, we used the same architecture as used in the pyramid scene parsing network (PSPNET) [27]. This provides pixel-level category prediction. To obtain the explicit relation between the feature vector and the feature of the local region of the food, the feature extraction module was trained in advance. It is possible to train this module by optimizing both ranking and food class; however, a dataset that has annotation of both rank and food class is not available. Therefore, we adopted a serial approach by pretraining the feature extraction layer with a large food class dataset and then connecting it to the ranking layer, followed by train ranking in an end-to-end manner.

**Figure 3:**
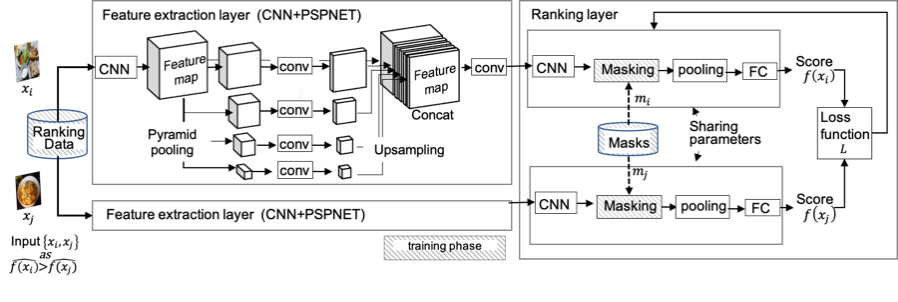
Proposed system for training ranking data of meals. The feature extraction layer consists of the convolutional neural network (CNN) and the same network as that of the pyramid scene parsing network (PSPNet) [27], which outputs pixel-wise feature maps. The ranking layer module is for estimating the scores. FC: fully convolutional layer.

We used the UEC FOOD-100 dataset [5,19], which includes 100 food categories, for pretraining the feature extraction layer. The network was pretrained to output the correct category of food. PSPNET [27] in the feature extraction layer was pretrained to the output food category by pixel. The entire architecture is shown in Figure 3.

Since the assertion of healthiness by the network has no meaning per se, we defined the deviation as the healthiness value, which was calculated as the distance from the mean value of the network output normalized by the variance. We call this variance the *healthiness score*, which is different from the *rank*.

### Experiments

#### Verifying the Accuracy of a Dietitian’s Subjective Evaluation

The expert (registered dietitian) had to infer ingredients and foods from the images.

We then confirmed the relation between the ranking based on viewing images with rankings according to the measured nutritional value in advance using the registered dietitian’s image-based rank to create an expanded ground truth set in our experiments.

#### Verifying the Accuracy of the Meal Rating Model

We conducted our experiment taking into consideration any errors in pair comparison and ranking estimation, unhealthy meal detection, and domain adaptability of meal images.

We used an original meal image database of patients with type 2 diabetes (see the General Ranking Data section below for the detailed process of ranking annotation) and the UEC Food Dataset [19]. We used 90% of the ranking data by the dietitian for training the proposed CNN-based ranking estimation system, and the rest of the ranking data were used for pair comparison/rank estimation and unhealthy meal detection.

For pretraining of the CNN and evaluation of the domain adaptability of the ranking estimate, we used the images in UEC Food Dataset.

#### Pair Comparison and Rank Estimation

We evaluated the accuracy of ranking healthiness under different conditions: (1) with and without pretraining the feature extraction layer, (2) with and without using a mask generated by semantic segmentation, (3) with CNN+FC (using a fully convolutional layer connecting CNN and Rank-SVM) or Rank-SVM as the output layer in the training and test phase, and (4) with rank-based CNN [21] using a mask.

We conducted experiments to examine the contribution of pretraining the CNN for feature extraction, an end-to-end structure using a CNN for the output layer compared to using Rank-SVM, and to examine the performance of our method compared to the original rank-based CNN proposed by Dubey et al [21]. Since the original rank-based CNN (RSS-CNN) does not have the structure of using a mask that indicates the region of food, we masked the region other than food in the input image by embedding 0 values to compare with our method in the same condition. We compared two networks: one based on RSS-CNN and the other based on Rank-SVM. The latter outputs the rank of healthiness by Rank-SVM using the CNN feature, which is generated by the same trained CNN as the network constructed from the RSS-CNN–based network.

The evaluation indices were the error rate of comparison *E_p_* and the average error of order *E_o_* determined as follows.

When the order relation between the estimated scores *s_i_* and *s_j_* of a pair of test images *I_i_* and *I_j_* are the same as the order relation between the pair of scores *ŝ_i_* and *ŝ_j_* assigned by the registered dietitian to *I_i_* and *I_j_*, the score estimation result for the pair *i* and *j* is counted as true, *C*(*i,j*)=1.

The accuracy of the ranking of pairs was calculated for all pairs of test images. The ratio of counts that proved true is then taken to be the error rate of comparison *E_p_* of the pairs.


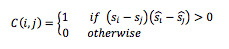



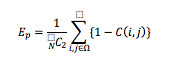


Where Ω is the set of all other pairs of test samples (*I_i_*, *I_j_*), (*i, j* ∈ {*I_1_, I_2_*,…, *I_N_*}) and *N* is the number of test samples.

We generated two rankings by sorting the test samples according to two scores: those estimated by the proposed method and those assigned by the registered dietitian. We then compared the order *o_i_* of *I_i_* to the order *ô_i_* by the registered dietitian, and calculated the error between *o_i_* and *ô_i_* as *e_i_*=∣*o_i_* – *ô_i_*∣; *o_i_* is obtained by sorting the images according to the estimated ranks.

The average error of order *E_o_* is taken to be the mean of *e_i_*; Ω is the set of test samples {*I_1_, I_2_*,…, *I_N_*} and *N* is the number of test samples.


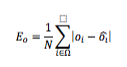


#### Unhealthy Meal Detection

The proposed system can be used to detect unhealthy meals. It can automatically identify which patients tend to select unhealthy meals and trigger oversight by health experts. To confirm this, we conducted an experiment on unhealthy meal detection.

#### Domain Adaptability of the Ranking Estimate

To test the domain adaptability of our method, we estimated the healthiness scores of the images of a publicly available database, UEC Food Dataset [5,19], whose domain is different from that of our training dataset.

## Results

### Dietitian Subjective Evaluation

**Figure 4:**
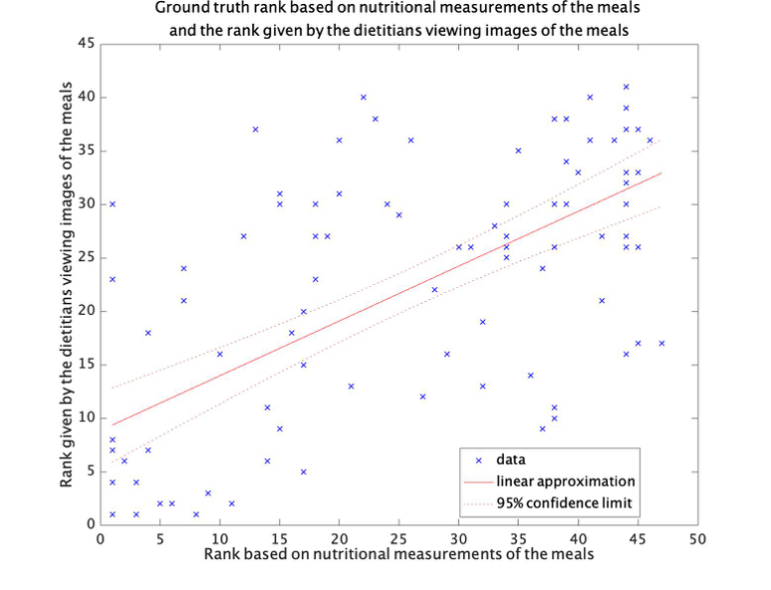
Ground truth rank based on nutritional measurements of the meals and the rank given by the dietitian viewing images of the meals.

Figure 4 shows the correlation between the rank judged based on viewing images and the ground truth rank. With a correlation coefficient of 0.73, we confirmed that ranking based on viewing images correlates with ranking based on the measured nutritional value. The Bland-Altman plot further confirmed that there is no fixed bias or proportional bias (Multimedia Appendix 2). Accordingly, we used a registered dietitian’s image-based rank to create an expanded ground truth set in our experiments.

### Pair Comparison and Rank Estimation

Table 1 shows the results of the accuracy achieved in ranking healthiness under different conditions. The result of the proposed method with masking, pretraining, and the CNN was better than that of the network without any module related to feature extraction.

**Table 1:** Evaluation of the error rate of healthiness between methods.

Method	Conditions for training the model			Error rate of the rank (*E*_o_)	Error rate of the pairwise rank (*E*_p_)
	Masking	Pretraining	Ranking method		
Proposed method	Yes	Yes	CNN^a^	13.94	16.40%
	Yes	No	CNN	14.59	17.16%
	No	Yes	CNN	16.6	19.5%
	Yes	Yes	Rank-SVM^b^	16.2	19.1%
Rank-based CNN [21]	Yes	No	CNN	15.44	18.15%

^a^CNN: convolutional neural network.

^b^SVM: support vector machine.

**Figure 5:**
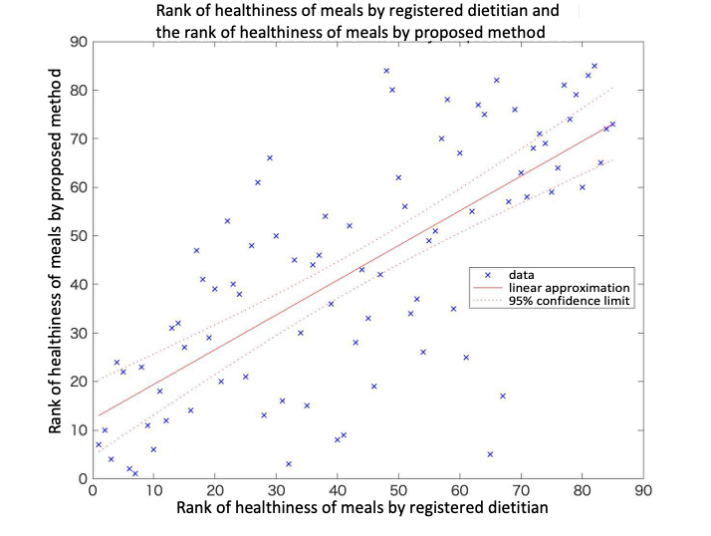
Comparison of the normalized ranking by the registered dietitian and the ranking estimates output by the proposed method.

The relation between the ranking of healthiness based on the dietitian’s judgment and the ranking estimated by the proposed method is shown in Figure 5. The rankings were normalized to have a mean of 0 and unit variance, and were then transformed to range from 0 to 100. The correlation coefficient between these rankings was 0.72. We also confirmed that there was no fixed bias or proportional bias by evaluation of the Bland-Altman plot (Multimedia Appendix 3).

### Unhealthy Meal Detection

Table 2 shows the results of the accuracy achieved in unhealthy meal detection by the proposed and rank-based CNN methods. Although the difference between methods was small, the proposed method with the full process was again superior.

**Table 2:** Evaluation of the accuracy of unhealthy meal detection.

Method	Conditions for training the model			Accuracy of unhealthy meal detection
	Masking	Pretraining	Ranking method	
Proposed method	Yes	Yes	CNN^a^	76.5%
	Yes	No	CNN	73.9%
	No	Yes	CNN	72.5%
	Yes	Yes	Rank-SVM^b^	70.3%
Rank-based CNN [21]	Yes	No	CNN	72.86%

^a^CNN: convolutional neural network.

^b^SVM: support vector machine.

**Figure 6:**
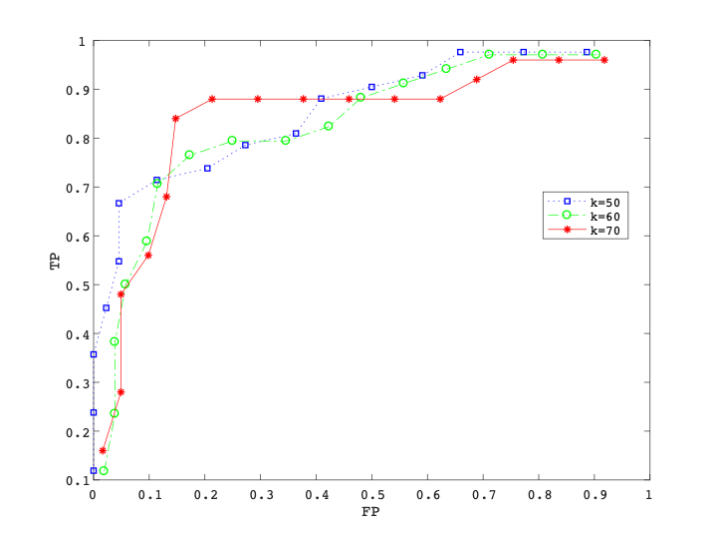
Receiver operating characteristic curves of unhealthy meal detection. The horizontal axis is the false positive rate (FP) and the vertical axis is the true positive rate (TP). Each curve shows the set of values of TP vs FP, when the definition of an unhealthy meal is a meal whose rank is lower than k% (k=50, 60, 70) of all meals.

The curves in Figure 6 show the rate in the number of meals that were detected as unhealthy but are actually healthy in reality versus that of meals detected as unhealthy and are unhealthy in reality. Each curve shows the values when “unhealthy” was defined as a meal ranking in the lower *k*% (*k*=50, 60, 70) of all meals.

Sample images and their healthiness values determined by the proposed method are shown in Multimedia Appendix 4, including images that have high or low scores, and examples of images with large error rates. These images show meals that were predicted to be very healthy yet deemed very unhealthy by the experts and vice versa.

### Domain Adaptability of Ranking Estimate

Multimedia Appendix 5 shows sample meal images from UEC-Food Dataset whose domain is different from that used in our training dataset, and the respective healthiness scores estimated by the proposed method, including meals that have higher and lower scores.

## Discussion

### Principal Findings

Our experimental results show that there is a relation between the ranking of healthiness based on the dietitian’s judgment and the ranking estimated by the proposed method (Figure 5), and it is possible that the CNN acquired something similar to human intuition. Multimedia Appendix 4 shows samples of meal images and the deviation values of healthiness as estimated by the proposed method. The meal images with high scores contained more dishes and more red or green colors than meal images with low scores. Although we cannot infer a cause from this finding, it is reasonable to suppose that that food color or the numbers of dishes in the image could have impacted the healthiness prediction.

In the case of images with large error rates (Multimedia Appendix 4), the red color of the raw meat or the yellow color of tempura in the left image may have induced the system to rate the dishes as healthy even though the dietitian judged them as unhealthy because of excessive calories. Conversely, the images below show meals that were predicted to be unhealthy but judged as healthy by the expert. The *yakisoba* dish in the left image consisted of stir-fried noodles with a lot of vegetables and meat; the dietitian judged this meal as healthy, but its color may have induced a negative prediction from the system. Since the logic of the prediction of healthiness by the network is not explicit, the impact of food color or number of dishes is uncertain. However, it appears that some implicit criteria formed by the dietitian had been transferred to the network model.

The images in Multimedia Appendix 5 show samples with scores that are relatively high or low in UEC Food Dataset, which uses a different domain from our original dataset in the training the model. Most of the meals with higher scores contained multiple dishes that have balanced, nutritious ingredients (ie, meat plus vegetables). The meals with lower scores are mainly single-plate dishes with no vegetables and high carbohydrates.

Although the meals were cooked at different times and included slightly different ingredients, some visual clues are associated with healthiness, and we can assume that the proposed system uses these visual clues in assessing healthiness.

In this work, we trained the network to learn a model based on the dietitian’s definition. It is possible to provide multiple indices in the future by establishing training data using our ranking GUI tool and training other models.

Based on pair comparison and rank estimation, we found that both masking and pretraining were effective methods to learn meal healthiness. The results of the end-to-end structure using a ranking layer were better than those obtained using a feature extraction layer and Rank-SVM. This suggests that the end-to-end approaches used by the ranking layer achieved better performance than feature-based extraction approaches used by Rank-SVM. The correlation coefficient of 0.72 between the rank of the proposed method and the rank given by the dietitian was not particularly high; however, this correlation and the error rate of pairwise comparison prediction of 16.4% (accuracy of 83.6%) are in line with previous work, including the accuracy of the original ranking method (ranking the safety of the city from its image) [21] of 73.5%, and a related study using machine learning–based calorie estimation from meal images that contain a single plate [20] that reported a correlation coefficient of the estimated calorie and the ground truth calorie of meal images of 0.78.

The result for unhealthy meal detection suggests that it is possible to set some appropriate thresholds that balance false positives and true positives. For example, when 60 meal images (2 meals from 30 users) are uploaded each day, by defining “unhealthy” as a meal ranking in the lower 50% of all meals, a threshold can be selected so that the lower 30 images are automatically detected with only a few healthy meals included. The assessment of domain adaptability of the ranking estimate suggests that our method has domain adaptability, so that meal images taken in various conditions (ie, at home or in restaurants) will be acceptable.

Since our meal image database consists of the photos of real meals of patients with type 2 diabetes, the scale of the database is not large, and the food categories are limited. In addition, the practically effective dataset for training the ranking model is even smaller since we allowed for tied ranks when the dietitian annotates the rank of the meal images. The number of dietitians giving a rank to part of the meal image database for training was also limited. Currently, we have data from two dietitians. In this work, we used the data from only one dietitian owing to the larger size of the dataset. Because of these limitations, the results of our experiments must be interpreted in light of the context of a pilot study. Generating a larger database with more categories of meals will help to improve the accuracy of ranking estimation.

We generated the model of ranking estimation using a machine-learning approach under the assumption that there is a relation between the appearance of meal images and the rank given by a dietitian. However, if a larger-scale dataset is available, there is a possibility that we will be able to classify meals into multiple categories for both healthy and unhealthy meals.

### Conclusions

We have presented an image-based system that can rank meals in terms of the overall healthiness of the dishes constituting the meal. First, we showed that the ranking has good correlation to nutritional value–based ranking. We then proposed a network that allows conditions that are important for judging the meal image, while extracting features that eliminate background information and are independent of location. Under these conditions, the experimental results showed that our network achieves higher accuracy of healthiness ranking estimation than the conventional image ranking method. Although the size of the training dataset is not yet sufficiently large for a training-only solution, introduction of pretraining of the feature extraction network using the food dataset enables the system to produce estimated rankings with high correlation to the ranking of an expert.

The results of this experiment in detecting unhealthy meals suggest that our system can be used to assist health care workers in establishing meal plans for diabetic patients who need advice in choosing healthy meals. Future work will include creating a larger dataset using the ranking data of multiple registered dietitians and improving the accuracy of inference.

## Appendix

Multimedia Appendix 1 Algorithm for generating the ranking dataset.

Multimedia Appendix 2 Bland-Altman plot of the ranks of healthiness of meals by the dietitian and the proposed method.

Multimedia Appendix 3 Bland-Altman plot of the mean value and the difference between the ground truth rank based on nutritional measurements and the rank given by the dietitian viewing images of the meals.

Multimedia Appendix 4 Samples of meal images and the deviation values of healthiness as estimated by the proposed method (top), and samples of meal images with large error rates (bottom).

Multimedia Appendix 5 Sample of meal images from UEC-Food Dataset and their healthiness scores estimated by the proposed method for meals with (a) higher scores and (b) lower scores.

## Abbreviations

CNN: convolutional neural network
MLRA: machine learning rank algorithm
PSPNET: pyramid scene parsing network
SVM: support vector machine
