# Supplementary material for: Machine Learning–Based Screening of Healthy Meals From Image Analysis: System Development and Pilot Study
Source: JMIR Form Res. 2020 Oct 26;4(10):e18507. doi: 10.2196/18507 (PMC7652690; doi:10.2196/18507)
Supplement: Multimedia Appendix 1 [file formative_v4i10e18507_app1.docx]

| Algorithm 1 |
| --- |
| 1. Procedure Ranking$\left( S \right)$ 2. Split S into half subset $S_{1}$ and $S_{2}$. 3. for $i=1,2$ do 4. if $n_{i}$ (numbers of samples of $S_{i}$) $\leq N_{c}$ then 5. Merge$\left( S_{i} \right)$. 6. else 7. Ranking$\left( S_{i} \right)$ 8. procedure Merge$\left( s_{i} \right)$ $\left( i=1,\cdots,k, k\geq1 \right)$ 9. if $\sum_{i} n_{i}$ (numbers of total samples) $\geq N_{c}$ then 10. Split $s_{i}$ into half subsets $s_{i1}$ and $s_{i2}$ . 11. Merge$\left( s_{i1} \right)$ 12. Merge$\left( s_{i2} \right)$ 13. else 14. Select a sample $p$ around the middle of sorted list as a pivot. 15. for $i=1$ to $k$ do 16. Add $p$ to $s_{i}$ . 17. Annotate rank manually on GUI and sort samples of $s_{i}$ . 18. Split $s_{i}$ into $l_{i}(s_{i},p)$ and $h_{i}(s_{i},p)$ by lower or higher than . 19. Store $l_{i}(s_{i},p)$ and $h_{i}(s_{i},p)$ in $L(s_{i},p)$ and $H(s_{i},p)$, each. 20. Merge$(L\left( s_{i},p \right))$ 21. Merge$(H\left( s_{i},p \right))$ 22. Concat $H\left( s_{i},p \right)$ and $L\left( s_{i},p \right)$ as $M\left( s_{i} \right)$ 23. return $M\left( s_{i} \right)$ |
