# Supplementary figures and images for: Machine Learning–Based Screening of Healthy Meals From Image Analysis: System Development and Pilot Study
Source: JMIR Form Res. 2020 Oct 26;4(10):e18507. doi: 10.2196/18507 (PMC7652690; doi:10.2196/18507)

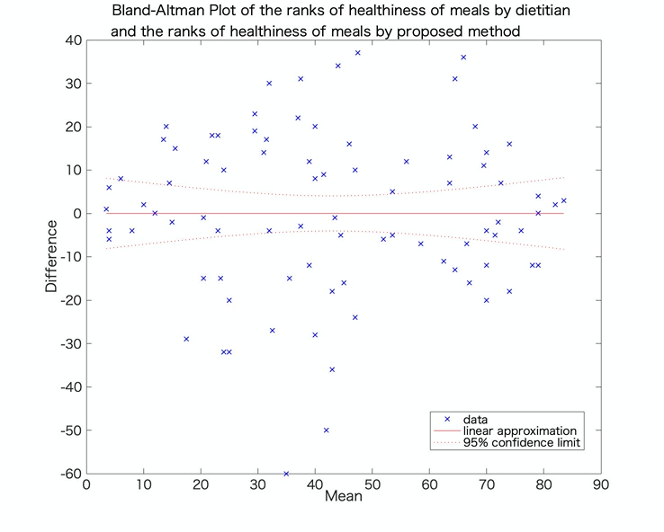

Supplement: Multimedia Appendix 2 [file formative_v4i10e18507_app2.png]

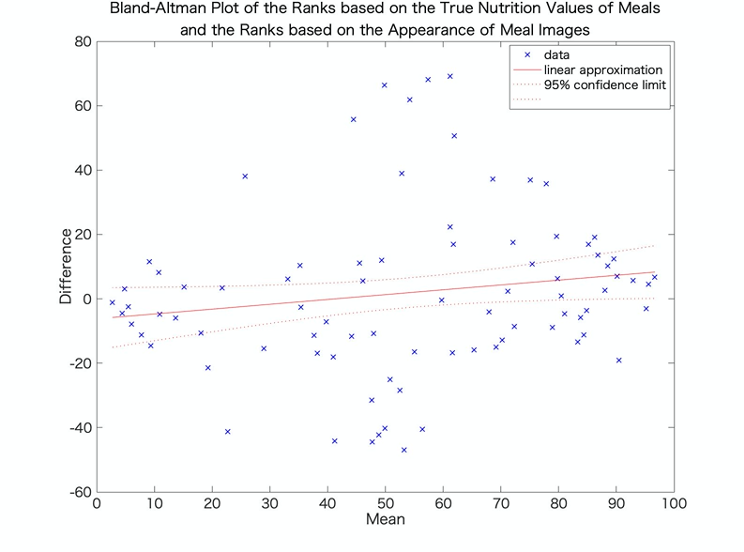

Supplement: Multimedia Appendix 3 [file formative_v4i10e18507_app3.png]
