# Supplementary material for: Machine Learning–Based Screening of Healthy Meals From Image Analysis: System Development and Pilot Study
Source: JMIR Form Res. 2020 Oct 26;4(10):e18507. doi: 10.2196/18507 (PMC7652690; doi:10.2196/18507)
Supplement: Multimedia Appendix 4 [file formative_v4i10e18507_app4.docx]

| **(a) Meal images that have high scores and the deviation values of healthiness.** | 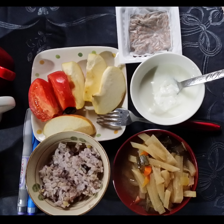 | 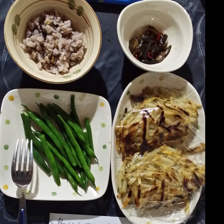 | 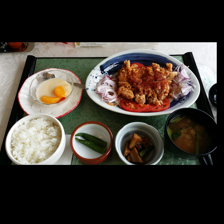 |
| --- | --- | --- | --- |
|  | 75 | 65 | 63 |
| **(b) Meal images that have low scores and the deviation values of healthiness.** | 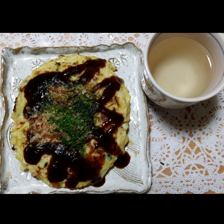 | 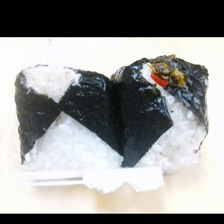 | 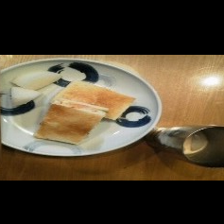 |
|  | 46 | 44 | 43 |

| **(a) Meal images that were estimated to be very healthy by the system but were rated as not very healthy by the registered dietitian (RD).** | 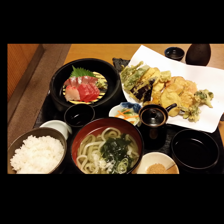 | 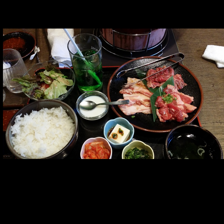 |
| --- | --- | --- |
|  | RD:5 (system: 66) | RD:17 (system:70) |
| **(b) Meal images that were estimated to be very unhealthy by the system but were rated as healthy by the registered dietitian (RD).** | 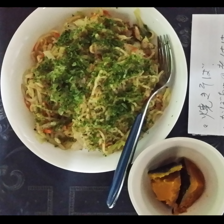 | 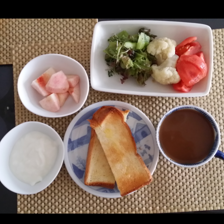 |
|  | RD:66 (system:44) | RD:61 (system:46) |
