# Supplementary material for: Machine Learning–Based Screening of Healthy Meals From Image Analysis: System Development and Pilot Study
Source: JMIR Form Res. 2020 Oct 26;4(10):e18507. doi: 10.2196/18507 (PMC7652690; doi:10.2196/18507)
Supplement: Multimedia Appendix 5 [file formative_v4i10e18507_app5.docx]

| **(a) Meal images that have high scores and the deviation values of healthiness in the UEC-Food dataset.** | 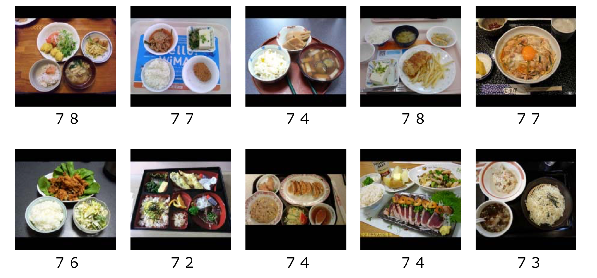 |
| --- | --- |
| **(b) Meal images that have low scores and the deviation values of healthiness in the UEC-Food dataset.** | 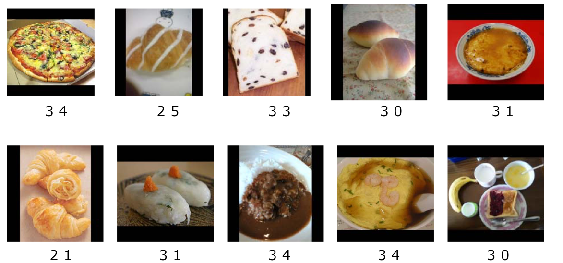 |
